# Identification of spinal tuberculosis subphenotypes using routine clinical data: a study based on unsupervised machine learning

**DOI:** 10.1080/07853890.2023.2249004

**Published:** 2023-08-23

**Authors:** Yuanlin Yao, Shaofeng Wu, Chong Liu, Chenxing Zhou, Jichong Zhu, Tianyou Chen, Chengqian Huang, Sitan Feng, Bin Zhang, Siling Wu, Fengzhi Ma, Lu Liu, Xinli Zhan

**Affiliations:** Department of Spine and Osteopathy Ward, The First Affiliated Hospital of Guangxi Medical University, Nanning, P.R. China

**Keywords:** Spinal tuberculosis, machine learning, cluster analysis, K-means, heterogeneity

## Abstract

**Objective:**

The identification of spinal tuberculosis subphenotypes is an integral component of precision medicine. However, we lack proper study models to identify subphenotypes in patients with spinal tuberculosis. Here we identified possible subphenotypes of spinal tuberculosis and compared their clinical results.

**Methods:**

A total of 422 patients with spinal tuberculosis who received surgical treatment were enrolled. Clustering analysis was performed using the K-means clustering algorithm and the routinely available clinical data collected from patients within 24 h after admission. Finally, the differences in clinical characteristics, surgical efficacy, and postoperative complications among the subphenotypes were compared.

**Results:**

Two subphenotypes of spinal tuberculosis were identified. Laboratory examination results revealed that the levels of more than one inflammatory index in cluster 2 were higher than those in cluster 1. In terms of disease severity, Cluster 2 showed a higher Oswestry Disability Index (ODI), a higher visual analysis scale (VAS) score, and a lower Japanese Orthopedic Association (JOA) score. In addition, in terms of postoperative outcomes, cluster 2 patients were more prone to complications, especially wound infections, and had a longer hospital stay.

**Conclusion:**

K-means clustering analysis based on conventional available clinical data can rapidly identify two subtypes of spinal tuberculosis with different clinical results. We believe this finding will help clinicians to rapidly and easily identify the subtypes of spinal tuberculosis at the bedside and become the cornerstone of individualized treatment strategies.

## 1. Introduction

Tuberculosis is still a global public health problem and poses a serious threat to human health [1]. Spinal tuberculosis accounts for around 50% of all bone and joint tuberculosis. It is one of the most severe and common extrapulmonary tuberculosis. As the disease progresses, bones are often severely damaged, causing scoliosis, affecting neural function, and severely affecting the quality of life of patients [2,3]. Unfortunately, a one-size fits all management and treatment approach is still implemented in clinical practice, which ignores the heterogeneity of spinal tuberculosis patients [4]. Inadequate treatment and management are one of the reasons for poor prognosis [5]. In addition, phenotypic heterogeneity is a major obstacle to tuberculosis management and personalized treatment. Completely understanding the inherent heterogeneity of tuberculosis is essential to formulate efficient intervention strategies [6]. Because the pathogenesis of spinal tuberculosis has not been elucidated, it is difficult to explain and predict the characteristics of patients with spinal tuberculosis.

Machine learning algorithms are widely used in clinical practice [7,8], especially in the diagnosis of tuberculosis [9]. Machine learning has shown strong effectiveness, as evidenced by the study conducted by Orjuela-Cañón et al. which indicates that ML algorithms can serve as effective diagnostic tools for tuberculosis, especially in settings with limited healthcare infrastructure [10]. Aguiar FS et al. have also developed models based on artificial neural networks for classifying hospitalized patients and risk allocation in environments with high tuberculosis prevalence [11]. And cluster analysis is a typical unsupervised machine learning method, which can effectively, accurately, and reasonably identify phenotypic heterogeneity according to the characteristics of patients’ diseases and classify heterogeneous queues [12]. Among these, the K-means clustering analysis is a good clustering method and is widely used in clinical practice [13,14]. For instance, Koo et al. successfully identified five phenotypes of pulmonary tuberculosis through K-means cluster analysis. Patients with these five phenotypes had significant differences in their symptoms and microbiological and radiological examination results. Thus this analysis provides a hierarchical medical method and has become the cornerstone of individualized treatment strategies [15]. In addition, K-means clustering analysis has been successfully applied to identify the subphenotypes of spinal tumors, sepsis, and other diseases [16,17]. However, no useful classification tool has been developed to identify the heterogeneity of spinal tuberculosis. Therefore, we proposed a K-means clustering method based on which only the routine available clinical data collected by patients within 24 h after admission can be used to identify subphenotypes of spinal tuberculosis. Finally, we compared the differences between the clusters in terms of clinical characteristics, surgical efficacy, and postoperative complications, and verified the accuracy of clustering.

## 2. Materials and methods

### 2.1. Patient

We reviewed and analyzed the perioperative clinical data of patients who received surgical treatment for spinal tuberculosis in the First Affiliated Hospital of Guangxi Medical University from June 2012 to June 2021. Inclusion criteria were [1] Clinical symptoms consistent with spinal tuberculosis: These encompass chronic back pain, progressive spinal deformity, weight loss, fatigue, and nocturnal sweating, etc [2]. Radiological manifestations consistent with spinal tuberculosis: These encompass vertebral body osteolysis, and the formation of abscesses, etc [3]. Lesions confirmed through percutaneous biopsy or postoperative pathological examination, showing pathological features of spinal tuberculosis such as caseous necrosis and granuloma, and further validated through culture to establish the presence of Mycobacterium tuberculosis [4], complete clinical data [5], no surgical history affecting the spine. The exclusion criteria were [1] pathological diagnosis after the operation is unclear [2], complicated with tumour or other immune-related diseases [3], incomplete clinical information, and [4] history of surgery affecting the spine. A total of 422 patients were included in the study (253 males and 169 females). In addition, the general information of patients, preoperative laboratory examination results, surgical conditions, postoperative complications, etc., were collected from the electronic medical record system. The study was approved by the Ethics Committee of the First Affiliated Hospital of Guangxi Medical University.

### 2.2. Data collection

General information about the patient collected included age, gender, body mass index (BMI), Oswestry Disability Index (ODI), Japanese Orthopedic Association (JOA) scores, and visual analog scale (VAS). Using the clinical data of patients, ODI, JOA, and VAS scores were jointly evaluated by two senior specialists for each patient. Patient’s laboratory test results, including C-reactive protein (CRP), erythrocyte sedimentation rate (ESR), white blood cells (WBC), haemoglobin, platelets, neutrophils, lymphocytes, monocytes, total protein (TP), albumin, monocyte count to lymphocyte count ratio (MLR), platelet count to monocyte count ratio (PMR), platelet count to lymphocyte count ratio (PLR), neutrophil count to lymphocyte count ratio (NLR), platelet count to neutrophil count ratio (PNR), C-reactive protein to albumin ratio (CAR), and Systemic Immune-Inflammation Index (SII) were collected. SII was calculated using the following formula: (neutrophil count × platelet count)/lymphocyte count [18]. Patients’ surgical data, including operation time (OT), bleeding volume (BV), blood transfusion, postoperative drainage volume (PDV), length of hospital stay (LOS) and postoperative complications, were collected. Postoperative complications were defined as surgical wound infections or systemic infections, internal fixation failures, thrombosis, respiratory failure, cerebrospinal fluid leakage, and other surgery-related diseases.

### 2.3. Cluster analysis

We performed the K-means cluster analysis based on the preoperative age, gender, BMI, WBC, haemoglobin, platelets, neutrophils, lymphocytes, monocytes, TP, albumin, ESR, MLR, PMR, PLR, NLR, PNR, SII and CAR of patients with spinal tuberculosis. K-means clustering can classify the data of unknown labels into different groups according to data characteristics. It is a clustering algorithm based on division, where each group of data is also called a “cluster,” and the center point of each cluster is called a “centroid.” The sample points close to the cluster centroid can be divided into the same cluster by calculating the Euclidean distance between the sample point and the cluster centroid [19]. The similarity between the two samples is measured by the Euclidean distance between them. As the distance between the two samples increases, it decreases the similarity between them [20]. Firstly, we used the Scale function in the “factoextra” package to standardize the data [21], and calculate the Hopkins statistics using the get cluster density function to evaluate the clustering trend of the dataset. Then perform K-means clustering analysis with the following specific steps [1]: K initial centroids are randomly selected, then calculate the distance from each sample point to the initial centroids and assign it to the nearest initial centroid. This will generate K clusters [2]. For each cluster, calculate the average distance of all sample points assigned to that cluster as the new centroid [3]. Repeat this process until the centroid positions remain unchanged. Finally, use the silhouette coefficient (SC) to find the optimal number of clusters (K value) [19,20]. The specific formula for calculating SC is as follows:

$$SC\left(i\right)=\frac{b\left(i\right)\;\;-\;\;a\left(i\right)}{\max\left[a\left(i\right),\;b\left(i\right)\right]\;}$$

In this formula, a(i) represents the average distance between the sample point and all other points in the same cluster, while b(i) represents the average distance between the sample point and all points in the next nearest cluster. For each cluster, the intra-cluster difference is small, while the inter-cluster difference is large, which is what the K-means clustering algorithm pursues, and SC is the key indicator to describe the intra-cluster and inter-cluster differences. From the formula, we can see that the value range of SC is (−1, 1). When SC approaches 1, the clustering effect is better; The closer it is to −1, the worse the clustering effect [22]. This process is achieved through the “Fpc” package. All processes are performed using the R software (version 4.2.1)

### 2.4. Statistical analysis

SPSS (IBM version 26.0) and R statistical software (version 4.2.1) were used for statistical analysis. A *t*-test or Mann–Whitney *U* test was used for continuous variables, and the chi-square test or Fisher’s exact test was used for categorical variables. Pearson’s test was used for correlation analysis of normally distributed data, whereas Spearman’s test was used for non-normally distributed data. For normally distributed continuous variables are expressed as mean ± standard deviation (SD). For non-normally distributed continuous variables are expressed as the median (percentiles). A *p* < 0.05 was defined as a statistical difference.

## 3. Result

### 3.1. Cluster analysis results

**Figure 1.**
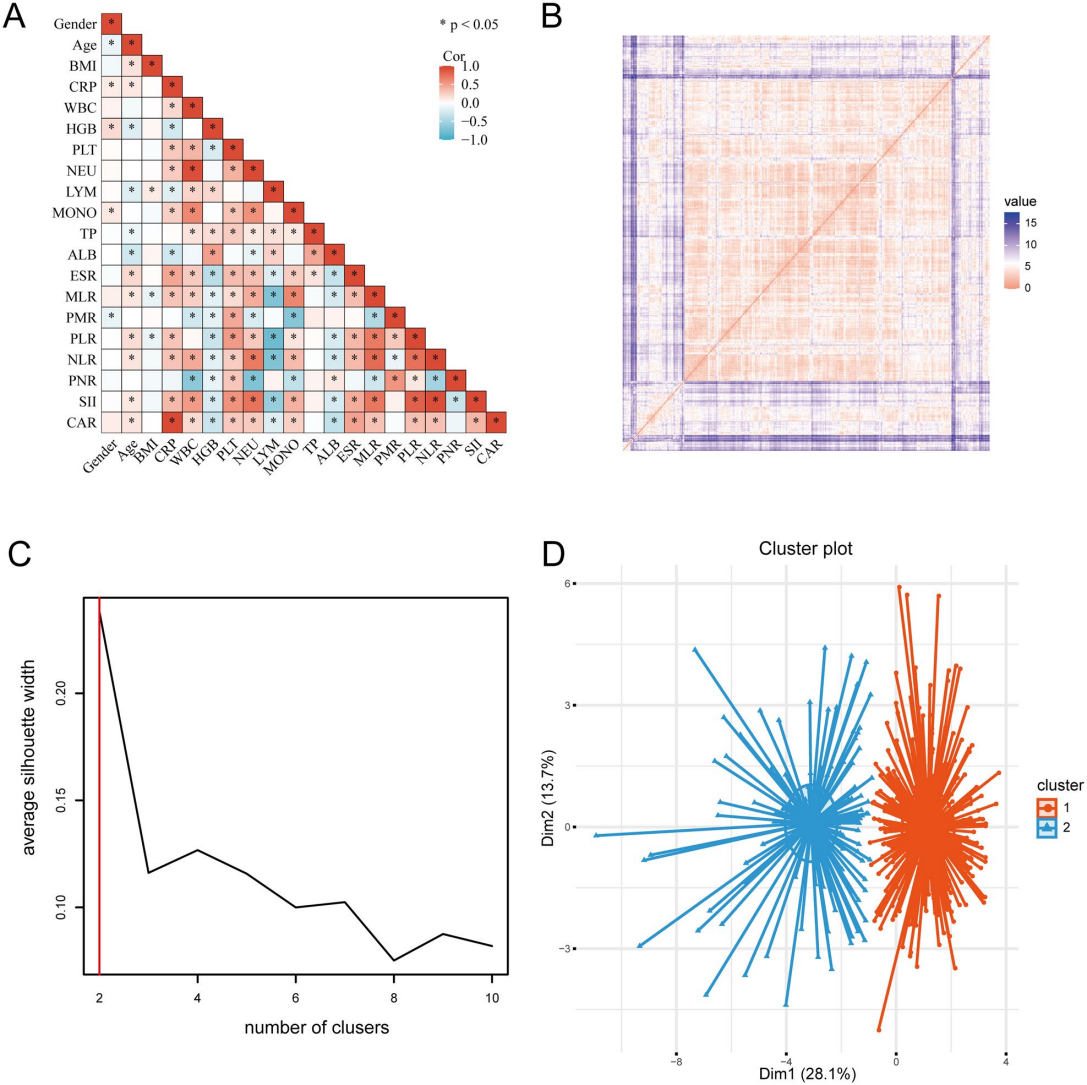
The process of K-means cluster analysis. **(A)** The correlation matrix. **(B)** The ordered dissimilarity matrix. **(C)** Optimal clustering number of the K-means clustering algorithm was determined by Silhouette coefficient (SC). **(D)** Scatter plots of patients’clinical data. Scatter points on the graph represent each patient, and the K-means clustering algorithm divides patients into two clusters.

To understand the correlation between variables, a correlation matrix (Figure 1(A)) was built to identify relationships between the variables, indicating that most variables have correlations between them. The cluster analysis results revealed the value of Hopkins statistics (0.815) and ordered dissimilarity matrix, which indicated that the dataset was significantly clusterable (Figure 1(B)). SC is a key indicator to describe the difference between inside and outside clusters. Through comparison, we found that when the clustering with *K* = 2 was found to have a higher Silhoutte score of 0.24, and the clustering effect was the best (Figure 1(C)). Therefore, 422 patients with spinal tuberculosis were finally clustered into clusters 1 and 2 (Figure 1(D)).

### 3.2. Studying patients’ characteristics by K-means clustering

**Figure 2.**
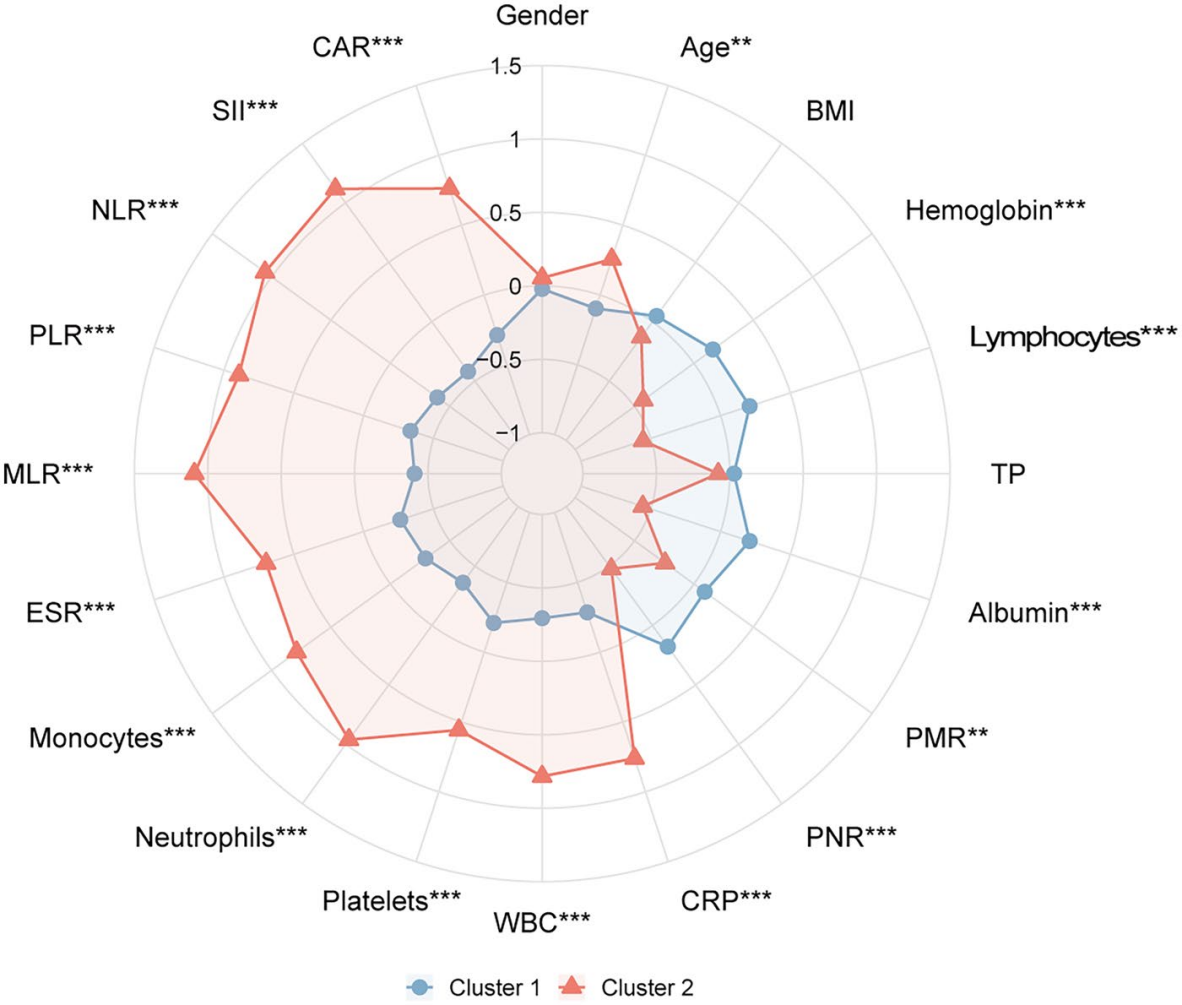
The radar chart of preoperative variables of spinal tuberculosis patients in two clusters. The K-means clustering algorithm normalized preoperative variables were compared between two clusters. Spoke lengths represent the average of each variable after the K-means clustering algorithm is normalized. Significance levels are presented with asterisks. **p-value < 0.01, ***p-value < 0.001.

A comparative analysis of preoperative variables between clusters revealed that the age of cluster 1 was lower than that of cluster 2 (*p* = 0.001). Haemoglobin, lymphocytes, albumin, PMR and PNR of cluster 1 were higher than those of cluster 2 (all *p* < 0.01). However, the CRP, WBC, platelets, neutrophils, monocytes, and ESR indexes of cluster 2 were higher than those of cluster 1 (all *p* < 0.001). In addition, the MLR, PLR, NLR, CAR, and SII indexes of cluster 2 were higher than those of cluster 1 (all *p* < 0.001). There was no significant difference in gender, BMI, and TP between the two clusters (all *p* > 0.05) (Table 1). The difference in preoperative variables between the two groups was well displayed on the radar chart (Figure 2).

**Table 1.** Baseline characteristics of the study patients by clusters.

Characteristics	Cluster 1 *n* = 307	Cluster 2 *n* = 115	*p*-value
Age	48.6 ± 17.37	54.7 ± 16.27	0.001
Gender			0.496
Male	181 58.96%	72 62.61%	
Female	126 41.04%	43 37.39%	
BMI	20.88 ± 4.14	20.2 ± 2.79	0.105
CRP	18.59 ± 23.83	58.98 ± 52.93	<0.001
ESR	34.39 ± 22.5	59.07 ± 25.12	<0.001
WBC	6.51 ± 1.95	9.57 ± 3.56	<0.001
Haemoglobin	122.3 ± 18	111.5 ± 17.32	<0.001
Platelets	283.4 ± 87.16	361.12 ± 114.78	<0.001
Neutrophils	3.92 ± 1.41	7.35 ± 3.31	<0.001
Lymphocytes	1.73 ± 0.8	1.13 ± 0.51	<0.001
Monocytes	0.56 ± 0.19	0.86 ± 0.33	<0.001
TP	70.89 ± 7.04	70.05 ± 9.45	0.388
Albumin	38.82 ± 4.75	34.84 ± 5.24	<0.001
MLR	0.37 ± 0.18	0.84 ± 0.33	<0.001
PMR	554.73 ± 239.62	474.98 ± 229.08	0.002
PLR	190.82 ± 90.76	363.6 ± 170.44	<0.001
NLR	2.59 ± 1.24	7.46 ± 4.49	<0.001
PNR	80.16 ± 36.29	56.97 ± 26.58	<0.001
SII	738.21 ± 405.94	2589.63 ± 1548.6	<0.001
CAR	0.5 ± 0.69	1.8 ± 1.77	<0.001

BMI: body mass index; CRP: C-reactive protein; ESR: erythrocyte sedimentation rate; WBC: white blood cells; TP: total protein; MLR: monocyte count to lymphocyte count ratio; PMR: platelet count to monocyte count ratio; PLR: platelet count to lymphocyte count ratio; NLR: neutrophil count to lymphocyte count ratio; PNR: platelet count to neutrophil count ratio; SII: systemic immune-inflammation index; CAR: C-reactive protein to albumin ratio.

### 3.3. Comparison of disease severity among clusters

**Figure 3.**
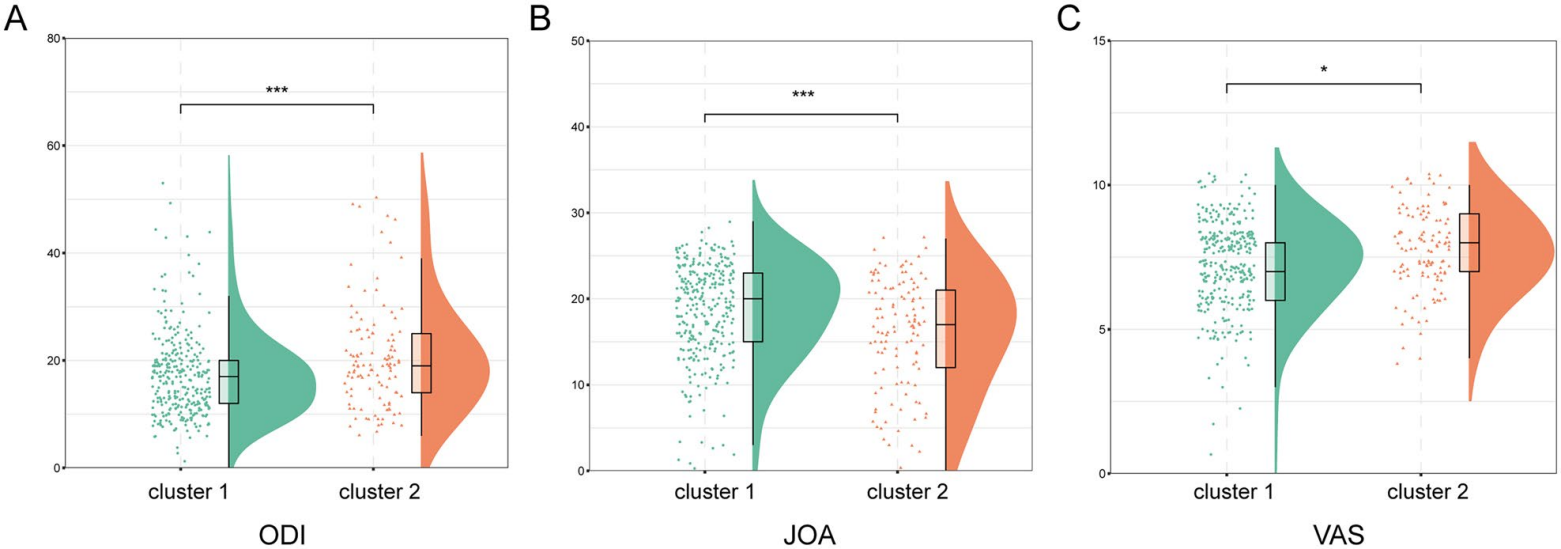
Comparison of disease severity between two clusters of spinal tuberculosis patients. **(A)** The differences in ODI scores between two clusters. **(B)** The differences in JOA scores between two clusters. **(C)** The differences in VAS scores between two clusters. *p-value < 0.05, ***p-value < 0.001.

**Figure 4.**
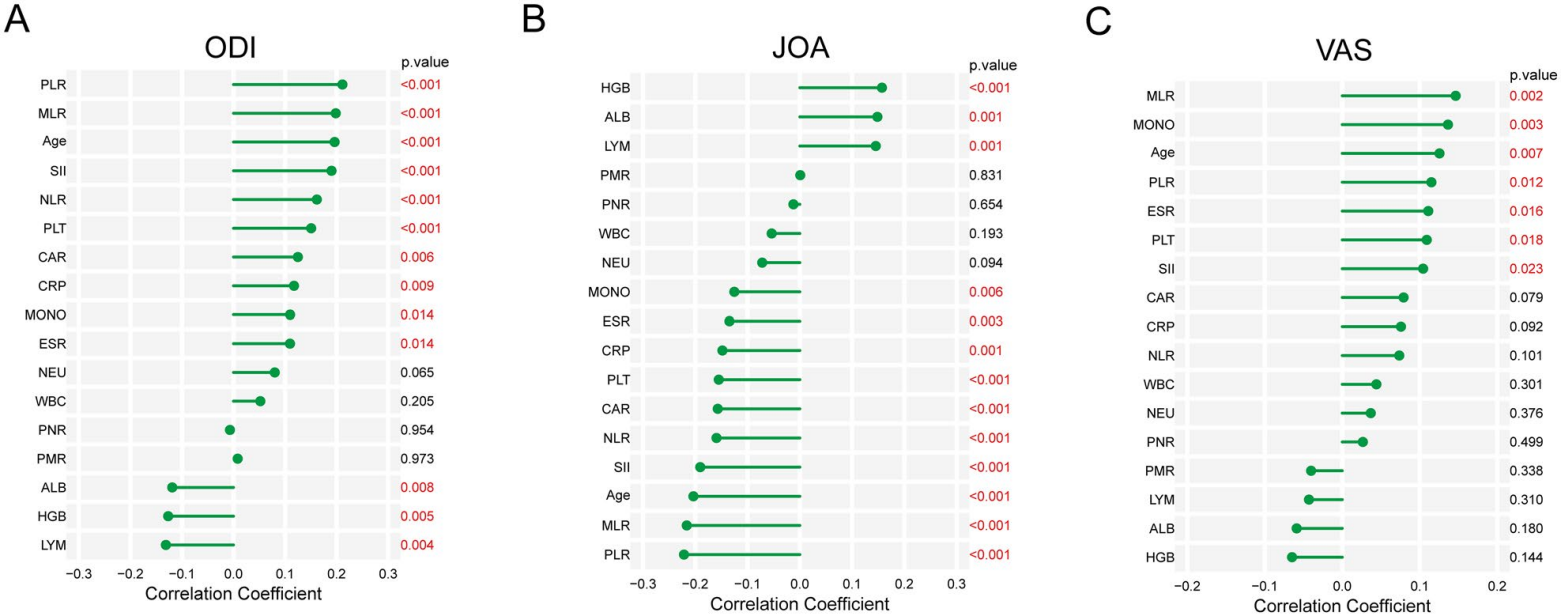
Correlation analysis between preoperative variables and disease severity. **(A)** Correlation between ODI score and preoperative variables. **(B)** Correlation between JOA score and preoperative variables. **(C)** Correlation between VAS score and preoperative variables.

A comparison and analysis of the scores of ODI, JOA, and VAS among clusters revealed found that the scores of ODI and VAS in cluster 2 were significantly higher than those in cluster 1 (*p* < 0.001 and *p* < 0.05), whereas the scores of JOA in cluster 1 were significantly higher than those in cluster 2 (*p* < 0.001) (Figure 3). It showed that cluster 2 had a higher disease severity. Correlation analysis revealed that multiple indicators were related to the severity of the disease. Among them, PLR, MLR, age, and SII had a strong positive correlation with ODI and VAS scores, and a strong negative correlation with JOA scores (all *p* < 0.05), indicating that age, PLR, MLR, and SII were positively related to the severity of the disease (Figure 4).

### 3.4. Comparison of surgical and postoperative variables among clusters

A comparison and analysis of the differences in surgical and postoperative variables between clusters revealed that the incidence of postoperative complications in cluster 2 was higher than that in cluster 1 (*p* < 0.05). Further analysis revealed that cluster 2 had a higher incidence of surgical wound infections than cluster 1 (*p* < 0.05). In addition, the hospitalization time of cluster 2 was longer than that of cluster 1 (*p* < 0.05) (Table 2).

**Table 2.** Postoperative conditions of two clusters of patients.

Postoperative conditions	Cluster 1 (*n* = 307)	Cluster 2 (*n* = 115)	*p*-value
Complication			0.028
Yes	74(24.10%)	40(34.78%)	
No	233(75.90%)	75(65.22%)	
Pulmonary infection			0.547
Yes	19(6.19%)	9(7.83%)	
No	288(93.81%)	106(92.17%)	
Hydrothorax			0.843
Yes	7(2.28%)	3(2.61%)	
No	300(97.72%)	112(97.39%)	
Wound infection			0.009
Yes	26(8.47%)	20(17.39%)	
No	281(91.53%)	95(82.61%)	
Gastrointestinal reaction			0.624
Yes	14(4.56%)	4(3.48%)	
No	293(95.44%)	111(96.52%)	
Thrombosis			0.714
Yes	4(1.30%)	1(0.87%)	
No	303(98.70%)	114(99.13%)	
Respiratory failure			0.937
Yes	5(1.63%)	2(1.74%)	
No	302(98.37%)	113(98.26%)	
Others			0.631
Yes	8(2.61%)	4(3.48%)	
No	299(97.39%)	111(96.52%)	

**Figure 5.**
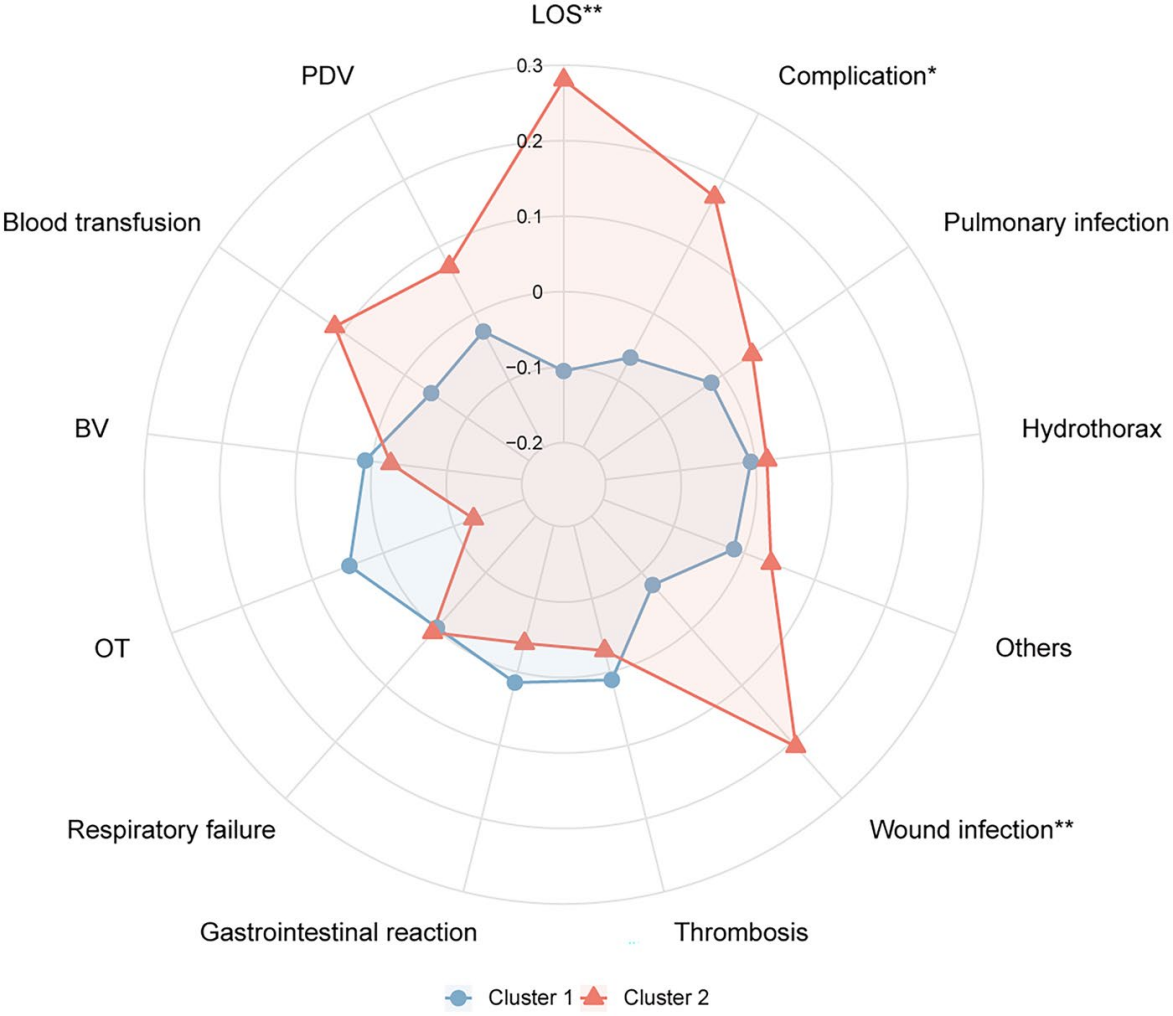
The radar chart of postoperative variables of spinal tuberculosis patients in two clusters. The K-means clustering algorithm normalized postoperative variables and were compared between two clusters. Spoke lengths represent the average of each variable after the K-means clustering algorithm is normalized. Significance levels are presented with asterisks. *p-value < 0.05, **p-value < 0.01.

The operative and postoperative variables, such as operation time, bleeding volume, blood transfusion, drainage volume, pulmonary infection, pleural effusion, gastrointestinal reaction, thrombosis, respiratory failure, and other complications, were similar among the clusters (*p* > 0.05) (Table 3). The radar map showed the differences in surgical and postoperative variables among clusters (Figure 5).

**Table 3.** Postoperative conditions of two clusters of patients.

Postoperative conditions	Cluster 1 (*n* = 307)	Cluster 2 (*n* = 115)	*p*-value
OT	149.52 ± 63.13	138.88 ± 52.36	0.108
BV	475.83 ± 458.99	460.26 ± 447.7	0.755
Blood transfusion			0.155
Yes	85(27.69%)	40(34.78%)	
No	222(72.31%)	75(65.22%)	
PDV	368.73 ± 283.73	397.31 ± 323.47	0.376
LOS	11.44 ± 5.8	14.33 ± 10.56	0.006

OT: operation time; BV: bleeding volume; PDV: postoperative drainage volume; LOS: length of hospital stay.

## 4. Discussion

Identification of different subphenotypes is a key component of personalized medicine. Identification of different subphenotypes of spinal tuberculosis will lead to better risk stratification and treatment decisions. However, one of the biggest challenges of subphenotype identification is how to translate research into clinical practice [14]. Therefore, we only used the patient’s age, gender, BMI, and 16 routinely available preoperative laboratory examination results as factors to ensure that the study adhered to clinical practice guidelines and had higher clinical significance. In addition, we could accurately identify the subphenotypes of spinal tuberculosis through K-means cluster analysis.

Clustering analysis is typical unsupervised learning, which can reveal the inherent properties of samples and the laws of their relationships. It is widely used in different fields, including clinical medicine and bioinformatics, one of which is used for disease classification [23]. Among several clustering analysis methods, K-means clustering is one of the commonly used clustering analysis algorithms [24] because it can maximize the separation of clusters and provide the largest range [25] for identifying different groups of patients. It has been successfully used to identify subtypes of sepsis [26], pulmonary tuberculosis [15], and cervical spondylotic myelopathy [27]. Therefore, we selected the K-means cluster analysis and successfully identified two phenotypes based on the conventional available natural characteristics of patients with spinal tuberculosis rather than prior knowledge, which enabled us to further study these characteristics and highlight those related to medical research assumptions. This method provides a more meaningful description and the distinction between patient groups in the queue [28]. Comparative analysis revealed that cluster 2 had higher disease severity. In the postoperative outcome, the incidence of complications in cluster 2 was significantly higher than that in cluster 1, especially wound infections and a longer hospital stay. In conclusion, this finding could be used as a significant reference for the prognosis stratification of patients with spinal tuberculosis in clinical practice.

ESR and CRP are commonly used indicators to evaluate the infection degree of inflammatory diseases [29]. A multicenter retrospective cohort study reported that the elderly and the increased ESR after treatment were the key factors for poor surgical prognosis of patients with spinal tuberculosis [30]. A study reported MLR as an inflammatory marker of tuberculosis, which is related to its severity [31]. Similarly, Chen et al. showed that MLR is an independent factor for the severity of spinal tuberculosis [32]. Monocytes can promote the release of inflammatory mediators after pathogen invasion. They transform into macrophages to participate in immune responses [33]. Research has shown that a low lymphocyte count is intricately related to inflammation [34], which could cause an MLR imbalance in inflammatory diseases. In addition, PLR and SII are important markers of inflammation which are significantly expressed in several diseases and are intricately related to the prognosis of diseases [35–37]. Albumin and haemoglobin are important nutrients for the human body [38]. Chen et al. reported that albumin is an important predictor of surgical site infection in patients with spinal tuberculosis. A lower albumin value is related to a higher risk of surgical site infections [39]. In the two sub-phenotypes identified, the level of age, CRP, ESR, monocytes, MLR, PLR, and SII in cluster 2 was significantly higher than that in cluster 1, whereas haemoglobin, lymphocytes and albumin were significantly lower than that in cluster 1. To summarize, patients in Cluster 2 had more serious diseases and worse prognoses than those in Cluster 1.

We used a classification method based on routinely available clinical data to further understand the subphenotypes of spinal tuberculosis. This can evaluate the severity of spinal tuberculosis and the differences in prognosis or treatment in clinical practice. However, this method for classifying patients with spinal tuberculosis requires additional external validation before its clinical implementation.

This study had several limitations: firstly, k-means is a widely used algorithm in different fields. However, it has some disadvantages such as being sensitive to outliers, hard-working with categorical variables, initialization issues, and election of number of the clusters, among others. Secondly, although we strive to minimize the potential impact of collinearity by standardizing the data. However, collinearity is still an issue that cannot be ignored, which may have an impact on the distance measurement between variables, thereby affecting the clustering results of the K-means algorithm. Thirdly, the sample size of this study was small and it was a single-center, retrospective study, which could have resulted in inevitable selection bias. In the future, the sample size should be increased and further verified by a multicenter, prospective study. In addition, the surgeon’s preferences and experience could affect the results of the study.

## Conclusion

K-means clustering analysis based on conventional available clinical data can rapidly identify two subtypes of spinal tuberculosis with different clinical results. We believe this finding will help clinicians rapidly and easily identify the subtypes of spinal tuberculosis at the bedside. Thus, it has the potential to become the cornerstone of individualized treatment strategies.

## Data Availability

The original contributions presented in the study are included in the article. Further inquiries can be directed to the corresponding author.
